# 

**Published:** 1845-07

**Authors:** 


TO READERS, CORRESPONDENTS, &c.

In addition, to our usual exchange list, we have received (in exchange,)

The Buffalo Medical Journal;

The Quarterly Summary of the Transactions of the College of Physicians, Philadelphia;

The American Quarterly Jour. of Agriculture and Science.

Also—Guthrie on the Anatomy and Diseases of the Urinary and Sexual Organs;

Esquirol on Insanity; (both from the publishers.)

The delay in the issue of the present number, has resulted from the absence of the editor. As he has returned and resumed his duties, no delay can again result from the same cause.

